# The Estimated Prevalence and Incidence of Endometriosis With the Korean National Health Insurance Service-National Sample Cohort (NHIS-NSC): A National Population-Based Study

**DOI:** 10.2188/jea.JE20200002

**Published:** 2021-12-05

**Authors:** Hyunkyung Kim, Minkyoung Lee, Hyejin Hwang, Youn-Jee Chung, Hyun-Hee Cho, Hyonjee Yoon, Mira Kim, Kyung-Hee Chae, Chai Young Jung, Sukil Kim, Mee-Ran Kim

**Affiliations:** 1 Department of Obstetrics and Gynecology, College of Medicine, The Catholic University of Korea, Seoul, Republic of Korea; 2 Department of Preventive Medicine, College of Medicine, The Catholic University of Korea, Seoul, Republic of Korea; 3 Biomedical Research Institute, Inha University Hospital, Incheon, Republic of Korea

**Keywords:** endometriosis, epidemiology, prevalence, incidence

## Abstract

**Background:**

The incidence and prevalence of endometriosis remain unclear due to diagnostic difficulties. Especially, there has been little information regarding the population-based epidemiology of endometriosis. The purpose of this study is to estimate the prevalence and incidence of endometriosis in Korea based on the health insurance claims data.

**Methods:**

This study is a retrospective cohort study using the Korean National Health Insurance Service-National Sample Cohort, which correspond to approximately 1 million Korean populations from 2002 to 2013. Patients aged 15–54 years were selected, and the prevalence and incidence of endometriosis were estimated by time and age groups.

**Results:**

The age-adjusted prevalence rate of endometriosis also increased from 2.12 per 1,000 persons (95% confidence interval [CI], 2.01–2.24) in 2002 to 3.56 per 1,000 persons (95% CI, 3.40–3.71) in 2013. The average adjusted incidence showed no statistically significant increase. However, the age-specific incidence of the 15–19 and 20–24 years age groups increased significantly from 0.24 and 1.29 per 1,000 persons in 2003 to 2.73 and 2.71 per 1,000 persons in 2013 (*R*^2^ = 0.93 and 0.77, *P* < 0.001), while the incidence rate of the age group 40–44 and 45–49 years decreased from 2.36 and 1.72 per 1,000 persons in 2003 to 0.81 and 0.27 per 1,000 persons in 2013 (*R*^2^ = 0.83 and 0.89, *P* < 0.001).

**Conclusion:**

The prevalence and incidence of endometriosis in Korean women were lower than that of previous reports in high-risk population studies. Furthermore, we found a significant increase in the diagnosis of endometriosis in younger age groups.

## INTRODUCTION

Endometriosis is a common benign disease characterized by the presence of endometrial glands and stroma outside the uterine cavity, mainly on the ovary, pelvic peritoneum, and rectovaginal septum.^1^ It is associated with infertility and various symptoms, such as dysmenorrhea, dyspareunia, non-cyclical pelvic pain, and non-gynecological cyclical pain.^2^ However, the intensity of symptoms is not always related to the stages of endometriosis, and some women can be asymptomatic.^3^^,^^4^ Diagnosis of endometriosis is mainly based on the symptoms, physical examination, and imaging techniques, such as vaginal ultrasound and magnetic resonance imaging (MRI). The gold standard of diagnosis is a visual inspection during surgery followed by a pathologic assessment.^5^

The prevalence and incidence of endometriosis are usually underestimated because of its difficulty in diagnosis. Until now, the overall prevalence was known to be about 10% in reproductive-age women and up to 50% of symptomatic women with infertility or pain in high-risk population.^6^^,^^7^ Still, there has been relatively little information in the literature regarding population-based epidemiology so far, especially among Asians.

The purpose of the study is to estimate the overall prevalence and incidence of endometriosis in Korea during the years 2002–2013 using a large sample of the national health insurance claims data and to describe the trend of the incidence rate of endometriosis according to time and age groups.

## METHODS

### Study data

The study data is the Korean National Health Insurance Service-National Sample Cohort (NHIS-NSC), which is a population-based cohort established by the National Health Insurance Service (NHIS). As the Korean NHIS is a universal coverage health insurance system, it includes public data on health care utilization, such as disease diagnoses, drug prescriptions, interventions, and procedures; health screening; socio-demographic variables; and mortality of the whole population of South Korea. The cohort database in this study is comprised of 1,025,340 participants who were randomly selected from the total 46,605,433 Korean population in January 1, 2002 and followed up for 11 years until December 31, 2013. According to prior study about the KNHIS-NSC, the data was built by systematic stratified random sampling with proportional allocation within each stratum using the individual’s total annual medical expenses as a target variable for sampling. First, 1,476 strata were constructed by age group, sex, participant’s eligibility status, and income level. Next, within each stratum, systematic sampling was conducted after sorting population data by the value of total annual medical expenses and maintaining a sampling rate of 2.2%. As this is a semi-dynamic cohort database, the cohort was refreshed annually by adding a representative sample of newborns to make up for the deceased or immigrants, sampled across 82 strata (two for sex, combined with 41 for parents’ income levels) using the 2.2% sampling rate.^8^ In 2013, the database included 1,014,730 participants.

### Case definition

Among the cohort participants, a total of 319,608 women aged 15–54 were selected from the NHIS cohort database in 2002. The diagnoses of endometriosis were coded using the standardized codes from the Korean version of the International Codes of Disease, 10th Edition (ICD-10). The ICD code of endometriosis is N80, N80.1, N80.2, N80.3, N80.4, N80.5, N80.6, N80.8, N80.9, except for adenomyosis (N80.0).

### Calculation of prevalence and incidence

The prevalence of endometriosis was calculated each year from 2002 to 2013. The denominator was the total number of women aged 15–54 as of December 31 each year. The nominator was the number of patients diagnosed with endometriosis among women aged 15–54 in the year. When the same ICD codes were found in a patient in a year, the case was counted as one. The age-adjusted prevalence was calculated using the standard population defined as the midyear Korean population data in 2002 from the Korean Statistical Information Service.^9^

The incidence case was defined as the first appearance of diagnostic codes of endometriosis in the health insurance claims regardless of hospital admissions or outpatient visits. To determine the previous history of endometriosis, the one year of look-back period was applied from the year 2003 because the patients would visit gynecologists within 1 year of the onset of endometriosis. Since the look-back period of each observation year increases by year and short look-back period is known to overestimate the incidence of diseases by misclassifying prevalent cases to incident cases, the prediction models on the proportion of misclassified incident cases were developed using multiple linear regression. The year of diagnosis and the number of patients were linearly related to the proportion of misclassification for endometriosis, and the look-back periods were logarithmically related to the proportion of misclassification. Using these findings, the following prediction model, which had the lowest root mean square error and highest estimated R-squared value, was developed and the estimated incidence of endometriosis was calculated each year using the following equation.^10^

$$\begin{array}{rl} \text{Estimated misclassification rate} & =-0.00522+0.000384\\ & \;\times \text{annual number of patients}\\ & \;+0.0457\\ & \;\times \ln(\text{look-back periods}). \end{array}$$

We used the actuarial method to calculate the incidence rate. The withdrawals, which were disqualified of the participants’ eligibility due to death or immigration, were assumed to occur at the midpoint of the study period, and hence W/2 was subtracted from the target population. The formula below was used to calculate the incidence rate of the year.

$${\hat{R}}_{\left(t_{0},t\right)}={\hat{CI}}_{\left(t_{0},t\right)}=\frac{I}{N_{0}^{t}-(\frac{W}{2})}$$

$\hat{R}$ = Estimated risk, $\hat{CI}$ = Estimated cumulative incidence, *I* = Number of incidence case, $N_{0}^{t}$ = Number of target population at start of follow-up, *W* = Number of withdrawals, *t*_0_,*t* = Given period

To estimate the risk for the accumulated period (*t*_0_,*t_j_*) in years, we combined the 1-year estimates of risk (*R_j_* = *CI_j_*) by using the following formula:

$${\hat{R}}_{\left(t_{0},t_{j}\right)}={\hat{CI}}_{\left(t_{0},t_{j}\right)}=1-\prod_{j=1}^{j}(1-{\hat{CI}}_{j})$$

The overall incidence rates and incidence rates by age group were calculated. The age-adjusted incidence rate was also calculated using the midyear Korean population data in 2002, as was the case with the method of calculating the age-adjusted prevalence. Additional lookback-adjusted incidence rates were also calculated and suggested. The rates were expressed as a number per 1,000 persons.

### Statistical analyses

All descriptive statistics were reported in numbers and percentages. 95 percent binomial confidence intervals (95% CIs) were suggested for both prevalence and incidence. SAS (version 9.2, Cary, NC, USA) was used as the statistical analysis tool. The statistical significance was set at *P* < 0.05.

### Statement of ethics

This data was restricted to those who had been given access by the NHIS. We applied for access to the NHIS with the study protocol, which was approved by the Institutional Review Board of principal investigator’s affiliation and approved by the NHIS (NHIS-2016-2-243). This study was approved by the Institutional Review Board of Seoul St. Mary’s Hospital (KIRB-0E513-001). Informed consent was not obtained because the data was already anonymized and de-identified by the NHIS before analysis.

## RESULTS

### Prevalence of endometriosis

Among approximately 1 million individuals from the NHIS cohort database, women aged 15–54 years were selected. While the number of reproductive-aged women decreased from 319,608 in 2002 to 302,760 in 2013, the number of women diagnosed with endometriosis had increased from 678 in 2002 to 1,052 in 2013 (Table 1).

**Table 1.** The crude prevalence rate of endometriosis in Korean women aged 15–54 years, 2002–2013

Age group	15–19 years		20–24 years		25–29 years		30–34 years		35–39 years		40–44 years		45–49 years		50–54 years		Total (15–54 years)	
	
	Case (per 1,000)	total	Case (per 1,000)	Total	Case (per 1,000)	Total	Case (per 1,000)	Total	Case (per 1,000)	Total	Case (per 1,000)	Total	Case (per 1,000)	Total	Case (per 1,000)	Total	Case (per 1,000)	Total
2002	7 (0.21)	33,948	40 (0.96)	41,841	95 (2.23)	42,651	157 (3.26)	48,143	126 (2.89)	43,645	117 (2.52)	46,458	91 (2.51)	36,223	45 (1.69)	26,699	678 (2.12)	319,608
2003	5 (0.16)	31,940	50 (01.21)	41,405	129 (3.27)	39,404	155 (3.32)	46,723	158 (3.55)	44,456	123 (2.69)	45,755	85 (2.24)	37,869	40 (1.49)	26,908	745 (2.37)	314,460
2004	6 (0.19)	31,682	52 (1.32)	39,484	124 (3.18)	38,973	161 (3.54)	45,491	142 (3.14)	45,266	137 (3.05)	44,977	109 (2.72)	40,127	37 (1.31)	28,153	768 (2.44)	314,153
2005	9 (0.29)	31,403	66 (1.76)	37,412	114 (2.92)	39,085	169 (3.83)	44,169	144 (3.17)	45,485	139 (3.17)	43,869	112 (2.69)	41,671	40 (1.29)	30,902	793 (2.53)	313,996
2006	9 (0.28)	31,587	52 (1.52)	34,159	118 (3.03)	39,002	158 (3.81)	41,486	157 (3.42)	45,927	145 (3.48)	41,627	131 (3.03)	43,204	33 (1.00)	33,014	803 (2.59)	310,006
2007	11 (0.33)	32,884	47 (1.42)	33,065	130 (3.23)	40,238	147 (3.60)	40,871	137 (2.95)	46,495	119 (2.82)	42,207	108 (2.41)	44,884	43 (1.23)	35,021	742 (2.35)	315,665
2008	4 (0.12)	32,877	34 (1.10)	30,801	116 (2.92)	39,706	138 (3.65)	37,774	152 (3.38)	44,945	134 (3.14)	42,630	117 (2.66)	43,957	30 (0.83)	36,179	725 (2.35)	308,869
2009	12 (0.36)	33,394	41 (1.35)	30,377	130 (3.44)	37,831	159 (4.27)	37,206	172 (3.93)	43,792	147 (3.38)	43,547	129 (2.99)	43,146	51 (1.33)	38,236	841 (2.73)	307,529
2010	11 (0.32)	34,126	54 (1.79)	30,212	155 (4.32)	35,861	169 (4.53)	37,277	199 (4.67)	42,628	165 (3.76)	43,929	140 (3.31)	42,339	70 (1.75)	40,083	963 (3.14)	306,455
2011	10 (0.30)	33,862	56 (1.82)	30,838	147 (4.36)	33,729	192 (5.03)	38,163	183 (4.48)	40,815	180 (4.00)	45,053	152 (3.73)	40,784	69 (1.63)	42,324	989 (3.24)	305,568
2012	14 (0.42)	33,525	64 (2.01)	31,762	140 (4.39)	31,899	191 (4.97)	38,458	181 (4.58)	39,548	189 (4.20)	45,046	160 (3.92)	40,783	61 (1.41)	43,385	1000 (3.29)	304,406
2013	20 (0.61)	32,875	62 (1.90)	32,626	131 (4.30)	30,473	236 (6.04)	39,055	178 (4.76)	37,372	198 (4.44)	44,602	144 (3.40)	42,297	83 (1.91)	43,460	1052 (3.47)	302,760

total	118 (0.30)	394,103	618 (1.49)	413,982	1,529 (3.41)	448,852	2,032 (4.11)	494,816	1,929 (3.71)	520,374	1,793 (3.38)	529,700	1,478 (2.97)	497,284	602 (1.42)	424,364	10,099 (2.71)	3,723,475

**Figure 1.**
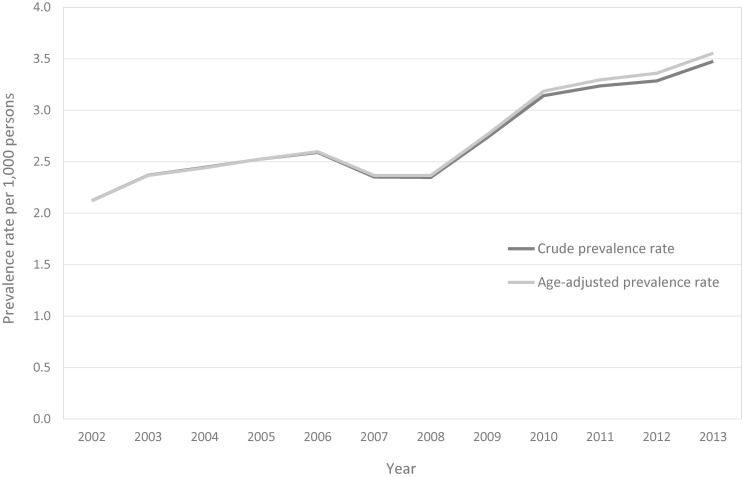
The age-adjusted prevalence rate (Gray) and crude prevalence rate (Black) of patients with endometriosis in Korea, 2002–2013

**Figure 2.**
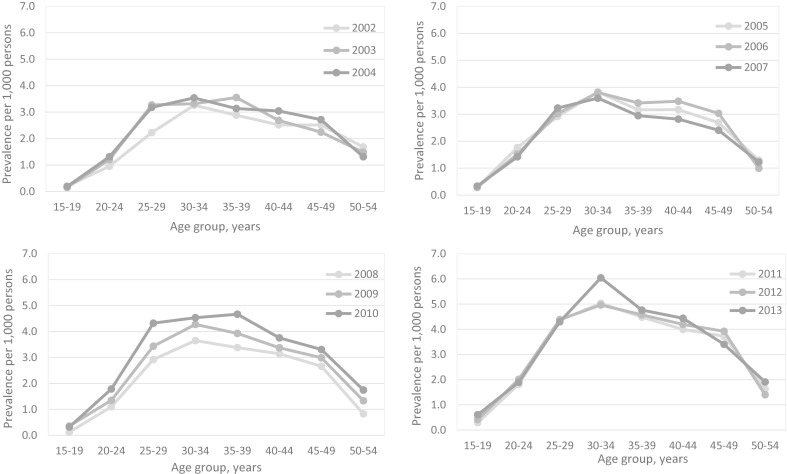
The age-specific prevalence rates of endometriosis in women in Korea, 2002–2013

The overall prevalence of endometriosis increased from 2.12 per 1,000 persons (95% CI, 2.11–2.13) in 2002 to 3.47 per 1,000 persons (95% CI, 3.44–3.51) in 2013. The age-adjusted prevalence rate of endometriosis also increased from 2.12 per 1,000 persons (95% CI, 2.01–2.24) in 2002 to 3.56 per 1,000 persons (95% CI, 3.40–3.71) in 2013. The prevalence of endometriosis temporarily decreased in 2007, but it continued to increase over the next 5 years (Figure 1). Regarding age-specific prevalence, the value increased sharply among women in their 20s, with the highest prevalence found in the 30–34 age group with 4.11 per 1,000 persons (95% CI, 4.07–4.14), and the value decreased as the age increased. The prevalence of women aged 30–34 years reached its peak at 6.04 per 1,000 persons (95% CI, 5.74–6.34) in 2013 (Figure 2).

### Incidence of endometriosis

**Figure 3.**
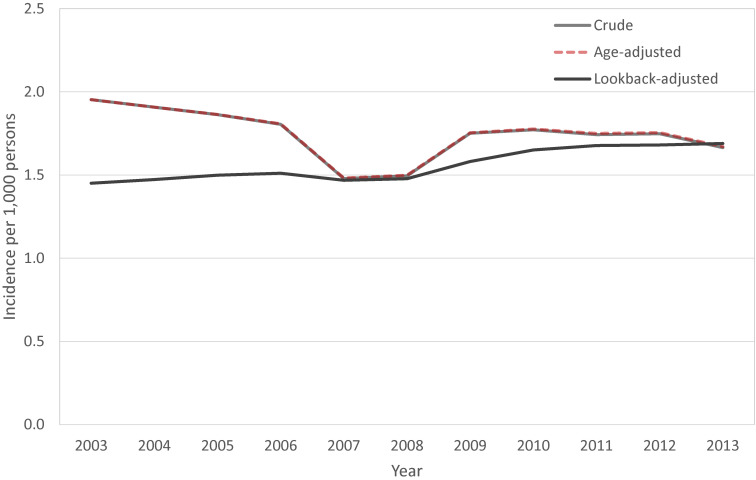
The crude annual incidence rate (Gray), age-adjusted incidence rate (Red dotted line) and lookback-adjusted incidence rate (Black) of endometriosis in Korean women, 2003–2013

A total of 5,908 new cases with endometriosis were identified after excluding patients with endometriosis in 2002. The crude annual incidence rate of endometriosis in persons aged 15–54 was 1.96 per 1,000 persons (95% CI, 1.95–1.96) in 2003, and the rate decreased to 1.67 per 1,000 persons (95% CI, 1.66–1.67) in 2013. The age-adjusted incidence rate of endometriosis was little different from the crude incidence rate, with the rate of 1.95 per 1,000 persons (95% CI, 1.85–2.07) in 2003, and to 1.67 per 1,000 persons (95% CI, 1.57–1.78) in 2013. The lowest incidence rate was observed in 2007 with 1.48 per 1,000 persons (95% CI, 1.39–1.58) (Figure 3).

After adjusting the misclassification, the annual incidence rate was 1.45 per 1,000 women (95% CI, 1.45–1.45) in 2003 and 1.69 per 1,000 persons (95% CI, 1.69–1.69) in 2013, but it was not statistically significant increase (*R*^2^ = 0.11, *P* = 0.17) (Figure 3). The difference between the crude annual incidence and lookback-adjusted annual incidence was 0.50 and 0.02 per 1,000 persons in 2003 and 2013, respectively.

**Figure 4.**
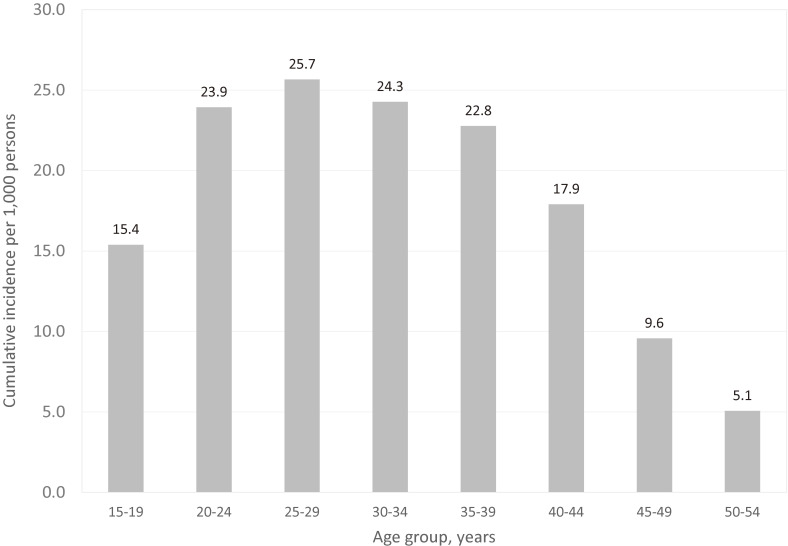
The 11-year cumulative incidence rate of endometriosis, by age group, 2003–2013

The cumulative incidence in women aged 15–54 during the study period between 2003 and 2013 was 19.07 per 1,000 persons, and the highest cumulative incidence was shown in the 25–29 age group with 25.66 per 1,000 persons. This result indicates that 25.66 out of 1,000 women aged 25–29 are at risk of being diagnosed with endometriosis newly within 11 years (Figure 4).

**Figure 5.**
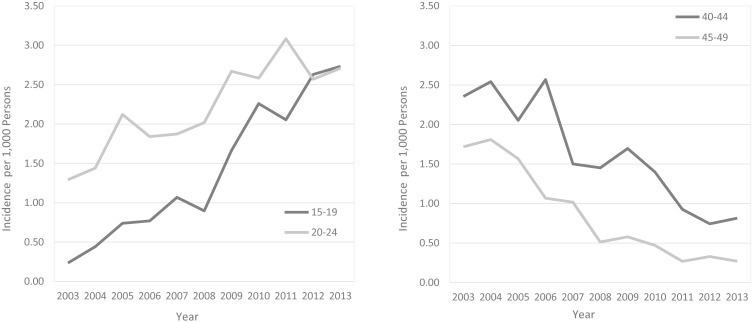
The annual incidence rate of endometriosis in the (Left) 15–24 years age group and (Right) 40–49 years age group, 2003–2013

Figure 5 shows the annual incidence rate of endometriosis in Korean women by age groups. The incidence rate significantly increased in the younger age group of patients under 25 years of age. The incidence rate of the age group 15–19 in 2003 was 0.24 per 1,000 persons (95% CI, 0.23–0.24), but it increased steadily to 2.73 per 1,000 persons (95% CI, 2.70–2.76) in 2013 (*R*^2^ = 0.93, *P* < 0.001). The incidence rate of the age group 20–24 also increased during the 11-year period from 1.29 per 1,000 persons (95% CI, 1.28–1.31) in 2003 to 2.71 per 1,000 persons (95% CI, 2.68–2.74) in 2013 (*R*^2^ = 0.77, *P* < 0.001). On the other hand, the incidence rate of women aged 40–49 decreased significantly. In 2003, the incidence rate of the age group 40–44 was 2.36 per 1,000 persons (95% CI, 2.33–2.38), but decreased to 0.81 per 1,000 persons (95% CI, 0.81–0.82) in 2013 (*R*^2^ = 0.83, *P* < 0.001). The incidence rate of the age group 45–49 also decreased from 1.72 per 1,000 persons (95% CI, 1.70–1.74) to 0.27 per 1,000 persons (95% CI, 0.27–0.27) in 11 years (*R*^2^ = 0.89, *P* < 0.001).

## DISCUSSION

We reported relatively low prevalence and incidence of endometriosis compared with those of previous studies.^6^^,^^7^^,^^11^^–^^19^ In a clinic or hospital-based setting, high prevalence and incidence of endometriosis among women in reproductive age have been reported, ranging from 2% to 50%.^6^^,^^7^^,^^12^^,^^13^^,^^18^ The reported prevalence and incidence were extremely heterogeneous due to different study settings and varying methodologies. Notably, the estimates in these studies may have been exaggerated because the studies included high-risk women with other gynecological conditions, such as subfertility and chronic pelvic pain.

The prevalence and incidence were slightly lower than or comparable to the rates of other population-based studies. In this study, the prevalence of endometriosis in Korean women aged 15–54 years was 3.7 per 1,000 persons in 2013, and the highest prevalence rate was observed among women aged 30–34 years with 6.04 per 1,000 persons. In Germany, the prevalence of endometriosis in the general population was estimated at 5.7 per 1,000 persons with the highest found in women aged 35–44 years.^19^ In the Unites States, based on the health insurance claims data, the prevalence was 0.7%, with the highest prevalence in the age group 30–39 years.^11^ In a recent study in Israel, the crude prevalence of endometriosis in 2015 was 10.8 per 1,000, and women aged 40–44 years had the highest prevalence rate of 18.6 per 1,000 persons.^15^

The adjusted annual incidence rate of endometriosis in this study was 1.45–1.69 per 1,000 persons. This incidence rate is comparable to that of Israel,^15^ Minnesota,^16^ Iceland,^20^ and Italy (range 0.72–1.87 per 1,000 persons)^21^ but lower than the rates from other previous population-based studies.^14^^,^^17^^,^^19^ Meanwhile, the age-specific annual incidence rates of endometriosis in population-based studies have not yet been sufficiently identified. In an Israeli study, the highest incidence rates were observed among women aged 25–39 years in 2015.^15^ In another study from Italy, the age-specific incidence of endometriosis was highest in the 31–35 year age group.^21^ Contrary to these findings, our study showed that the incidence rate of endometriosis has increased sharply in the young age over 11 years, highest being in the age group 15–24 in 2013.

There are several reasons for the low prevalence and incidence rate of our study. First, the patient could have remained undiagnosed because the endometriosis is coded based on the doctor’s record. As definite diagnosis of endometriosis requires visual inspection of the pelvic cavity, the doctor may have been reluctant to document the diagnosis of endometriosis before the confirmation by operation.^20^ Also, it is likely that the women with deep infiltrating endometriosis (DIE) were excluded from the patient group due to the difficulty in diagnosis by only clinical and imaging examination.^22^ Second, as the previous studies were performed using databases of regional health insurance or health care service,^11^^,^^15^^,^^19^ these studies could not represent the entire national population. In the studies using data from regional health insurance or health care service, more patients may have been diagnosed with endometriosis due to their higher frequencies of visits to gynecologists.^11^ Third, the cultural reasons for reluctance to take oral contraceptives in Korean women^23^ may also contribute to the low prevalence and incidence rate of endometriosis. In fact, according to data released by the United Nations in 2015, the rate of Korean women taking oral contraceptives is significantly lower than in other countries.^24^ Recent study of Chapron, et al revealed that past use of oral contraceptives is associated with endometriosis, especially DIE,^25^ although the relationship between the use of oral contraceptives and endometriosis remains still controversial. For additional reasons, the true prevalence and incidence of endometriosis in Korean women may be lower than in women in other countries. Several studies suggested the racial and ethnic differences in endometriosis that Asian women were at higher risk of endometriosis compared with women of other races.^26^^,^^27^ However, more recent prospective cohort study of the Nurses Health Study II found no significant difference in prevalence of endometriosis between race and ethnicity.^17^ As this is the first large epidemiological study of Asian women with endometriosis, further study is needed to compare racial and ethnic differences in the prevalence and incidence of endometriosis.

In this study, the annual incidence rate of newly diagnosed endometriosis in women aged 15–54 years varied little throughout the 11-year period. Considering that the birth rate of Korean women from 2.8 in 1980 to 1.19 in 2013,^28^ we expected a sharp increase in the annual incidence rate of endometriosis. According to the published studies, the risk of endometriosis has an inverse association with parity of two or more children.^14^^,^^26^^,^^29^^,^^30^ However, the crude annual incidence of endometriosis seemed to decrease, and there was only a small increase of annual incidence after the look-back adjustment of misclassification rate, even without statistical significance. In fact, we found that Korean ICD code (N80) was subdivided according to the disease location after 2007. Therefore, it is likely that the misclassified diagnostic code for adenomyosis (N80.0) was included in endometriosis diagnoses prior to 2007 in this study.

Another significant finding in this study is that the incidence rate has increased steeply in the younger age groups, while that of women aged over 40 has decreased significantly. This trend may be explained by the decreased menarche age of Korean women from 13.4 years in 2001 to 12.4 years in 2011.^31^ Previous studies have reported that early menarche age increases the risk of endometriosis because the early exposure to estrogen caused by early menarche age may increase the risk of endometriosis.^14^^,^^29^^,^^32^^,^^33^ In addition, the increased maternal age at first birth in Korean women from 28.3 years in 2002 to 30.7 years in 2013 could be an another factor in the increase incidence rate in the younger age group.^34^ An increased number of exposures to menstruation due to higher maternal age at first birth may have affected the risk of endometriosis in women in their 20s. Also, given that cumulative incidence rate for 11 years was the highest in their 20s and endometriosis is the cause of subfertility, early detection of endometriosis in younger age group is important for fertility preservation. As a result of the increased incidence of endometriosis in young aged women, the proportion of women first diagnosed with endometriosis in their 40s would have tended to decrease relatively.

The present study has several methodological limitations. First, the prevalence and incidence rate may have been underestimated, for endometriosis could be asymptomatic for a long period before visiting a hospital. Also, doctors have tended not to document the diagnosis if the patient was asymptomatic or had another primary diagnosis. In addition, we used the endometriosis diagnosis code based on the claim data. Although the classical diagnosis of endometriosis requires surgical visualization, this study included patients who were clinically suspicious with endometriosis as well as the patients confirmed by surgical interventions. While the diagnosis of endometriosis has recently been gradually shifting on a clinical basis, there has been no validation study using clinical diagnosis to detect the patient with endometriosis. Still, considering that gynecologists directly provide the primary health care services in Korean health care system and use ultrasound devices to diagnose diseases even in their private clinics, the diagnostic accuracy of endometriosis would be higher in Korean data than in other countries. The validation study of endometriosis using ICD codes based on clinical diagnosis is planned later.

Nevertheless, the strength of the present study is that it is the largest population-based study with a long observation period of 12 years. So far, the epidemiology of many hospital-based studies has had a significant gap in population-based studies, and there are only few national level studies in the world. Several previous large population-based studies were performed using databases of regional health insurance or during short follow-up period. The NHIS-NSC database in this study contains representative large-scaled population-based cohort data, since it is based on nationwide health insurance data generated by public institutions’ involvement.^8^ This database includes not only all information of hospitals and private clinics, but also personal identification numbers, which enabled us to find multiple visits to the doctors and to determine the exact year of the first diagnosis of endometriosis, which is important for analysis of the incidence rate. In addition, as long follow-up periods reveal the disease-free period more precisely, more accurate analyses could be made.

This study is the first large-scale epidemiological study of Asian women with endometriosis. We reviewed 65 articles via a search of PubMed including studies from 2000 to 2017, and only one study from Japan reported the prevalence of endometriosis in Asian (6.8%), including only 15,019 women.^35^ Given that there may be differences between race, ethnicity, and geography in pathogenesis of endometriosis, this study could be an important cornerstone for further follow-up studies.

Through this study, we identified the epidemiology of endometriosis in reproductive-aged women of South Korea. The prevalence and incidence of endometriosis were lower than that of previous reports in high-risk population studies but comparable with other population-based studies. Furthermore, we found a significant trend towards an increase in the diagnosis of endometriosis in younger age groups among Korean women. Considering that endometriosis is associated with subfertility, early detection and proper treatment of endometriosis in younger age groups are essential to improve the capacity of fertility.

## APPENDIX A. SUPPLEMENTARY DATA

eTable 1. The annual incidence rate and cumulative incidence rate of endometriosis, by age group, 2003–2013
