# Supplementary material for: The Estimated Prevalence and Incidence of Endometriosis With the Korean National Health Insurance Service-National Sample Cohort (NHIS-NSC): A National Population-Based Study
Source: J Epidemiol. 2021 Dec 5;31(12):593–600. doi: 10.2188/jea.JE20200002 (PMC8593577; doi:10.2188/jea.JE20200002)
Supplement: Supplementary file 1 [file je-31-593-s001.pdf]

eTable 1. The annual incidence rate and cumulative incidence rate of endometriosis, by age group, 2003-2013

Age group	Year	Duration	Pupulation	incident case	Withdrawal case	Annual incidence	Cummulative incidence	
	j	(t(j-1), t(j))	N	I(j)	W(j)	R(j)	R(i(0)~i(j))	
15–19	0	(0,0)	33,948	7	0			1
	1	(0,1)	33,941	8	28	0.00024	0.00024	0.9997642
	2	(1,2)	33,905	15	44	0.00044	0.00068	0.999321604
	3	(2,3)	33,846	25	48	0.00074	0.00142	0.998582942
	4	(3,4)	33,773	26	45	0.00077	0.00219	0.997813674
	5	(4,5)	33,702	36	42	0.00107	0.00325	0.996747159
	6	(5,6)	33,624	30	425	0.00090	0.00415	0.995852186
	7	(6,7)	33,169	55	232	0.00166	0.00580	0.994195093
	8	(7,8)	32,882	73	1,157	0.00226	0.00805	0.991948394
	9	(8,9)	31,652	65	17	0.00205	0.01009	0.989910799
	10	(9,10)	31,570	83	11	0.00263	0.01269	0.987307793
	11	(10,11)	31,476	86	10	0.00273	0.01539	0.984609802
20–24	0	(0,0)	41,841	40	0			1
	1	(0,1)	41,801	54	55	0.00129	0.00129	0.998707314
	2	(1,2)	41,692	60	90	0.00144	0.00273	0.997268497
	3	(2,3)	41,542	88	104	0.00212	0.00485	0.995153297
	4	(3,4)	41,350	76	104	0.00184	0.00668	0.993321934
	5	(4,5)	41,170	77	130	0.00187	0.00854	0.991461192
	6	(5,6)	40,963	82	636	0.00202	0.01054	0.98946095
	7	(6,7)	40,245	107	327	0.00267	0.01318	0.986819524
	8	(7,8)	39,811	101	1,422	0.00258	0.01573	0.984270451
	9	(8,9)	38,288	118	19	0.00308	0.01876	0.981236269

	10	(9,10)	38,151	98	14	0.00257	0.02128	0.978715266
	11	(10,11)	38,039	103	14	0.00271	0.02394	0.976064665
25–29	0	(0,0)	42,651	95	0			1
	1	(0,1)	42,556	114	102	0.00268	0.00268	0.997317963
	2	(1,2)	42,340	107	115	0.00253	0.00521	0.994794152
	3	(2,3)	42,118	92	140	0.00219	0.00738	0.992617567
	4	(3,4)	41,886	107	142	0.00256	0.00992	0.990077567
	5	(4,5)	41,637	92	137	0.00221	0.01211	0.987886313
	6	(5,6)	41,408	80	635	0.00195	0.01404	0.985962976
	7	(6,7)	40,693	83	282	0.00205	0.01606	0.983944951
	8	(7,8)	40,328	105	1,107	0.00264	0.01865	0.981347452
	9	(8,9)	39,116	89	24	0.00228	0.02089	0.979113923
	10	(9,10)	39,003	94	25	0.00241	0.02325	0.976753433
	11	(10,11)	38,884	96	18	0.00247	0.02566	0.974341385
30–34	0	(0,0)	48,143	157	0			1
	1	(0,1)	47,986	129	125	0.00269	0.00269	0.99730821
	2	(1,2)	47,732	131	123	0.00275	0.00543	0.994567576
	3	(2,3)	47,478	118	145	0.00249	0.00791	0.992091936
	4	(3,4)	47,215	101	144	0.00214	0.01003	0.98996646
	5	(4,5)	46,970	84	155	0.00179	0.01181	0.988193103
	6	(5,6)	46,731	105	753	0.00227	0.01405	0.985954695
	7	(6,7)	45,873	91	255	0.00199	0.01601	0.983993368
	8	(7,8)	45,527	94	804	0.00208	0.01806	0.981943609
	9	(8,9)	44,629	93	25	0.00208	0.02010	0.979896816
	10	(9,10)	44,511	97	28	0.00218	0.02224	0.977760717
	11	(10,11)	44,386	92	27	0.00207	0.02427	0.97573347
35–39	0	(0,0)	43,645	126	0			1

	1	(0,1)	43,519	114	105	0.00262	0.00262	0.997377291
	2	(1,2)	43,300	91	138	0.00210	0.00472	0.99527784
	3	(2,3)	43,071	105	130	0.00244	0.00715	0.99284785
	4	(3,4)	42,836	93	146	0.00217	0.00931	0.990688627
	5	(4,5)	42,597	68	122	0.00160	0.01090	0.989104867
	6	(5,6)	42,407	81	747	0.00193	0.01280	0.987198827
	7	(6,7)	41,579	90	223	0.00217	0.01494	0.985056236
	8	(7,8)	41,266	83	796	0.00203	0.01694	0.983055657
	9	(8,9)	40,387	94	46	0.00233	0.01923	0.98076631
	10	(9,10)	40,247	90	43	0.00224	0.02143	0.978571956
	11	(10,11)	40,114	55	38	0.00137	0.02277	0.977229608
40–44	0	(0,0)	46,458	117	0			1
	1	(0,1)	46,341	109	119	0.00236	0.00236	0.997644847
	2	(1,2)	46,113	117	135	0.00254	0.00489	0.995109867
	3	(2,3)	45,861	94	151	0.00205	0.00693	0.993066855
	4	(3,4)	45,616	117	132	0.00257	0.00948	0.990516057
	5	(4,5)	45,367	68	130	0.00150	0.01097	0.989029256
	6	(5,6)	45,169	65	762	0.00145	0.01241	0.987593896
	7	(6,7)	44,342	75	247	0.00170	0.01408	0.985918815
	8	(7,8)	44,020	61	767	0.00140	0.01546	0.984540587
	9	(8,9)	43,192	40	70	0.00093	0.01637	0.983628067
	10	(9,10)	43,082	32	52	0.00074	0.01710	0.982897017
	11	(10,11)	42,998	35	60	0.00081	0.01790	0.982096389
45–49	0	(0,0)	36,223	91	0			1
	1	(0,1)	36,132	62	84	0.00172	0.00172	0.998282073
	2	(1,2)	35,986	65	108	0.00181	0.00352	0.996476208
	3	(2,3)	35,813	56	112	0.00157	0.00508	0.9949156

	4	(3,4)	35,645	38	134	0.00107	0.00615	0.993852954
	5	(4,5)	35,473	36	123	0.00102	0.00716	0.992842585
	6	(5,6)	35,314	18	611	0.00051	0.00767	0.992332104
	7	(6,7)	34,685	20	244	0.00058	0.00824	0.991757887
	8	(7,8)	34,421	16	874	0.00047	0.00871	0.991290958
	9	(8,9)	33,531	9	83	0.00027	0.00898	0.991024558
	10	(9,10)	33,439	11	67	0.00033	0.00930	0.990698226
	11	(10,11)	33,361	9	76	0.00027	0.00957	0.990430655
50–54	0	(0,0)	26,699	45	0			1
	1	(0,1)	26,654	33	54	0.00124	0.00124	0.998760656
	2	(1,2)	26,567	20	136	0.00075	0.00199	0.998006846
	3	(2,3)	26,411	11	110	0.00042	0.00241	0.997590316
	4	(3,4)	26,290	10	107	0.00038	0.00279	0.997210086
	5	(4,5)	26,173	2	130	0.00008	0.00287	0.997133695
	6	(5,6)	26,041	5	426	0.00019	0.00306	0.996940661
	7	(6,7)	25,610	15	228	0.00059	0.00365	0.996354134
	8	(7,8)	25,367	5	691	0.00020	0.00384	0.996155034
	9	(8,9)	24,671	7	79	0.00028	0.00413	0.995871938
	10	(9,10)	24,585	10	90	0.00041	0.00453	0.995466122
	11	(10,11)	24,485	13	77	0.00053	0.00506	0.99493676
All	0	(0,0)	319,608	678				1
	1	(0,1)	318,930	623	672	0.00196	0.00196	0.998044533
	2	(1,2)	317,635	606	889	0.00191	0.00386	0.996137745
	3	(2,3)	316,140	589	940	0.00187	0.00572	0.994279079
	4	(3,4)	314,611	568	954	0.00181	0.00752	0.992481278
	5	(4,5)	313,089	463	969	0.00148	0.00899	0.991011309
	6	(5,6)	311,657	466	4,995	0.00151	0.01048	0.989517545

7	(6,7)	306,196	536	2,038	0.00176	0.01222	0.987779599
8	(7,8)	303,622	538	7,618	0.00179	0.01399	0.986007076
9	(8,9)	295,466	515	363	0.00174	0.01571	0.9842874
10	(9,10)	294,588	515	330	0.00175	0.01743	0.9825657
11	(10,11)	293,743	489	320	0.00167	0.01907	0.980929111
